# The full mitochondrial genomes of Mangalica pig breeds and their possible origin

**DOI:** 10.1080/23802359.2017.1390415

**Published:** 2017-10-17

**Authors:** Krisztián Frank, János Molnár, Endre Barta, Ferenc Marincs

**Affiliations:** Agricultural Biotechnology Institute, National Agricultural Research and Innovation Centre, Gödöllő, Hungary

**Keywords:** Mangalica pig, turopolje breed, next generation sequencing, mitogenome, breed history

## Abstract

The mitogenomes of one animal of each of the three Mangalica breeds, Blonde, Red, and Swallow-belly were assembled from reads obtained by Next Generation Sequencing of the three genomes. Features of the mitogenomes were identical in the three breeds, apart from a second *tRNA-Val* gene on the L strand in Swallow-belly. Phylogenetic comparison of the three mitogenomes with 112 full mtDNA sequences clearly put Mangalicas into the European clade. Comparing the mitogenome of eight Mangalica animals revealed particular differences between them. The mitogenome of some Mangalicas was closely related to the Croatian Turopolje breed and this indicates either the common origin of their maternal lineages or admixture of some populations of the breeds. However, the origin of the mitogenome of certain purebred Mangalicas kept in the Hungarian Mangalica Gene Reserve still remains unknown.

## Introduction

The three fatty-type Mangalica pig breeds, Blonde, Red, and Swallow-belly, are farmed in growing number in Hungary and also kept in several other European and overseas countries (Marincs et al. 2013) for up-market pork production. They have a quite complex history (Bökönyi 1974; Egerszegi et al. 2003), which goes back to the late 1700s, early 1800s, when their spontaneous and conscious breeding started involving the old Serbian pig breeds Sumadia (Sumadinka) and Syrmian (sometimes also called Black Mangalica) and three, already extinct, Carpathian Basin pig races, Bakonyi, Szalontai, and Alföldi, which might be originated from old domesticated pigs brought to the Carpathian Basin by ancient Romans.

Previously, we have studied the relationship between maternal lineages of Mangalica and other pig breeds (Marincs et al. 2013). The results indicated that 197 out of the 203 examined Mangalica individuals carried the ancient ANC-Aside D-loop signature (Larson et al. 2007). One hundred and twenty-two Mangalicas belonged to three Mangalica-specific D-loop haplotypes, while 64 animals belonged to two major European haplotypes (Marincs et al. 2013).

In this study, we present the mitogenome of the three Mangalica breeds assembled form next-generation-sequencing data and investigate the molecular phylogenetics of full mtDNA sequences of different pig breeds in order to determine a presumptive relatedness of maternal lineages of Mangalica pigs.

## Materials and methods

To assemble the mitogenome of the three Mangalica and a Duroc animal, 100 bp Illumina reads obtained from genome sequencing of one male animal of each bread (Molnár et al. 2013) were separately aligned to the AJ002189 (Ursing and Arnason 1998) pig mtDNA reference sequence using the Burrows-Wheeler Alignment Tool (Li and Durbin 2009). The mitochondrial sequences were assembled from the aligned reads by using SAMtools (Li et al. 2009).

Full mitochondrial nucleotide sequences of domestic pig (*Sus scrofa*) breeds were downloaded from the National Center for Biotechnology Information website. The mitochondrial genome of bearded pig (*Sus barbatus*) was also downloaded to use as outgroup. The H strand sequences of the mitogenomes were aligned using ClustalW2 (Larkin et al. 2007) and manually adjusted in a few instances. Phylogenetic analyses were performed by a Bayesian approach using BEAST version 1.8.2 (Drummond et al. 2012). Maximum likelihood analysis was carried out with the bootstrap test, with 1000 replicates, using complete deletion for gaps/missing data, with the general time reversible model. Bayesian Markov chain Monte Carlo (MCMC) sampling was performed for 10 000 000 iterations using estimated model parameters as starting values. The phylogenetic tree was drawn from the 50% majority rule consensus of trees sampled every 1000 generations. Sequence alignments were performed using the software Geneious v.9.1.7 (geneious.com).

Mitogenomes of this study were deposited into the NCBI Nucleotide database under accession numbers MF183222 (Blonde), MF183223 (Red), MF183224 (Swallow-belly) and MF183225 (Duroc).

## Results and discussion

Genome-sequencing of one male individual of each of the Blonde, Red, and Swallow-belly Mangalica breeds (Molnár et al. 2014) provided 260,270, 98,832 and 104,478 reads that aligned to the pig reference mitochondrial genome (Ursing and Arnason 1998), resulting in 1,571×, 602×, and 638× coverage, respectively. All three mitogenomes contained two ribosomal RNA and thirteen protein genes, the Blonde and Red individuals carried 22 tRNA genes, while the Swallow-belly animal contained 23 tRNA genes due to the presence of a second *tRNA-Val* gene on the L-strand (Table 1). Most of the genes were encoded on the H-strand.

**Table 1.** Features of the mitogenomes of the 4S, 15F and 13V Mangalica pigs.

	Position					
Gene	Start	End	Length	Start codon	End codon	Strand
*trnF*	1	70	70			H
*rrnS*	71	1030	960			H
*trnV*	1032	1099	68			H
*rrnL*	1100	2667	1568			H
*trnL*	2668	2742	75			H
*nad1*	2745	3698	954	ATG	TAG	H
*trnI*	3700	3768	69			H
*trnQ*	3766	3838	73			L
*trnM*	3840	3909	70			H
*nad2*	3910	4950	1041	ATT	TAG	H
*trnW*	4952	5019	68			H
*trnA*	5026	5093	68			L
*trnN*	5095	5169	75			L
*trnC*	5202	5267	66			L
*trnY*	5267	5332	66			L
*cox1*	5334	6875	1542	ATG	TAA	H
*trnK*	6882	6950	69			L
*trnD*	6958	7025	68			H
*cox2*	7026	7712	687	ATG	T	H
*trnT*	7714	7780	67			H
*atp8*	7782	7982	201	ATG	TAA	H
*atp6*	7943	8620	678	ATG	TAA	H
*cox3*	8623	9405	783	ATG	TA	H
*trnG*	9407	9475	69			H
*nad3*	9476	9820	345	ATA	TA	H
*trnR*	9823	9891	69			H
*nad41*	9892	10185	294	GTG	TAA	H
*nad4*	10182	11558	1377	ATG	T	H
*trnV*	11559	11627	69			L
*trnH*	11560	11628	69			H
*trnS*	11629	11687	59			H
*trnL*	11688	11757	70			H
*nad5*	11758	13575	1818	ATA	TAA	H
*nad6*	13565	14089	525	ATG	TAA	L
*trnE*	14090	14158	69			L
*cob*	14163	15314	1152	ATG	TA	H
*trnT*	15303	15370	68			H
*trnP*	15370	15434	65			L
*D-loop*	15434	16679	1245			

Previously we have studied the relationship between Mangalica and other pig breeds by comparing amplified and sequenced partial mitochondrial D-loop sequences (Marincs et al. 2013; Molnár et al. 2013). Such D-loop sequences, however, often differ in length, because the primer pairs used for D-loop amplification usually vary from study to study. Those partial sequences, therefore, should have been truncated to the same length for comparison (Marincs et al. 2013), which obviously results in loss of information. To overcome this limitation, in this study we used the full mitogenomes of 115 pig individuals of a number of different breeds (Supplementary Table S1) to investigate the phylogenetic relationship between Mangalica and other breeds.

**Figure 1.**
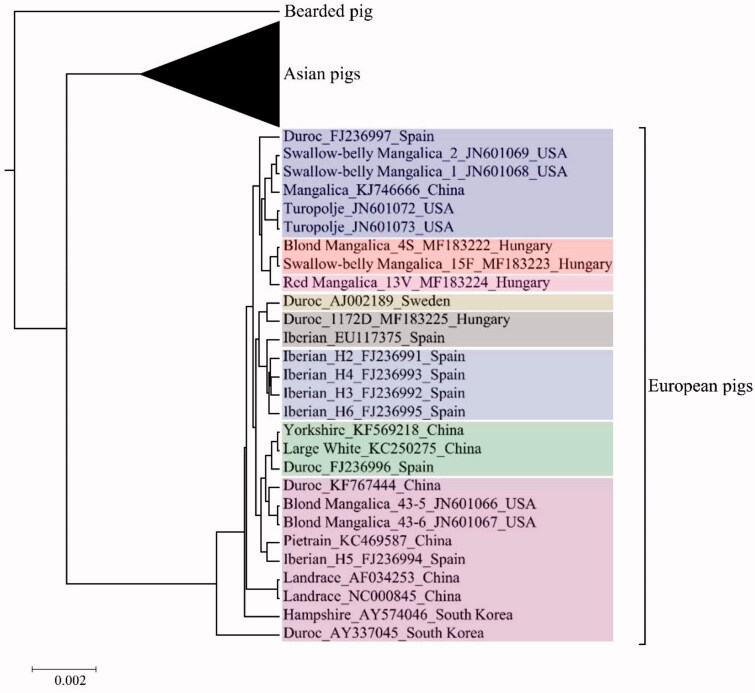
Phylogenetic tree showing the relationship between the mitogenomes of the three genome-sequenced Mangalica individuals (Molnár et al. 2014) and other European breeds. Individuals are labelled by their NCBI accession number and ID (if any). Colors indicate the D-loop haplotype of the animals (refer to Supplementary Fig. 4), according to Marincs et al. (2013); HAP13, HAP16, HAP45, HAP149, NEW, HAP07, HAP44, HAP08. Scale bar represents branch length (substitution per site).

There was a clear separation between the European and Asian breeds in the phylogenetic tree (Supplementary Fig. S1), as expected, although some Berkshire and Yorkshire individuals were placed into the Asian clade, indicating the introgression of maternal lineages of Asian breeds into their bloodline. Within the European branch (Figure 1), six Mangalica and two Turopolje individuals formed a well-separated clade, although the three genome-sequenced Mangalica individuals (Blonde, 4S; Swallow-belly, 15F; Red, 13V; Molnár et al. 2014) formed a separate branch within the clade. The Mangalica/Turopolje clade also contained two Swallow-belly Mangalicas and two Turopolje pigs, of which mitogenomes were sequenced by Cannon et al. (2015) and a Mangalica without breed specification (KJ746666). Interestingly, another two Blonde Mangalica individuals (Cannon et al. 2015) were positioned into a separate clade containing non-Mangalica breeds too (Figure 1).

The Mangalicas 4S, 15F and 13V with assembled mitogenomes, were kept at the Hungarian Mangalica Gene Reserve in the village of Emőd, Hungary, and represent the bloodlines “Bátor” (♀) and “Bácska” (♂), “Szank” (♀) and “Csatár” (♂), and “Szolnok” (♀) and “Mecsér” (♂), respectively. The Mangalicas studied in the USA (Cannon et al. 2015) were imported directly from Austria (Terry Brandebourg, personal communication), but their lineages were not revealed. According to another study though, most of the Austrian Mangalicas were imported from Hungary (Druml et al. 2012), thus it is possible that the USA Mangalicas have Hungarian origin and are not from the separated Mangalica population that remained in Austria after the break-up of the Austro-Hungarian Empire following World War I. The origin and breed of the Mangalica sequenced in China is not known.

To compare the Mangalica mitogenomes, their sequences were aligned, which revealed certain differences between them. In addition to the five one-base-pair deletions being present in different animals, the Mangalica of China and the Hungarian Mangalicas have a 50 and a 90 bp deletion, respectively, compared to the USA Mangalica pigs (Supplementary Fig. S2). Regarding SNPs, the Mangalica of China has the highest number of differences compared to every other individual, and the number of SNPs is higher between the Hungarian and USA Mangalica groups than within each group (Supplementary Fig. S3).

Previously we have determined the D-loop haplotype of 2713 pig individuals belonging to five cosmopolitan breeds, 38 local breeds from nine and wild boars from 14 European countries, respectively (Marincs et al. 2013). *In silico* haplotyping of the Mangalica individuals in this study revealed that Mangalicas 4S and 15F carry haplotype HAP16, while 13V carries haplotype HAP45 (Supplementary Table S2). Amongst the haplotypes, which were identified in domesticated pigs (Marincs et al. 2013), both HAP16 and HAP45 were considered Mangalica-specific since no individuals of other local European and Cosmopolitan breeds carried those, although HAP45 was found in five Austrian wild boar individuals. HAP16 and HAP45 were the fifth and third most abundant haplotypes amongst the Mangalicas included in that study. In contrast, the Blonde and Swallow-belly Mangalicas sequenced in the USA belonged to haplotypes HAP08 and HAP13 (Supplementary Table S2), respectively, which were the first and fourth most abundant haplotypes amongst all individuals (Marincs et al. 2013). HAP08 was found in 132 cosmopolitan pigs, and in 314 and 63 local pigs and wild boars representing eight and six countries, respectively, (Marincs et al. 2013). HAP13 was found in 35 cosmopolitan animals, and in 70 and 69 local pigs and wild boars, which originated from four and eight countries, respectively, (Marincs et al. 2013). The Chinese Mangalica also belonged to haplotype HAP08 (Supplementary Table S2). It is worth to note here that the clades of the European part of the phylogenetic tree (Figure 1.) were consistent with the D-loop haplotypes of the individual pigs (Supplementary Fig. S4).

Based on D-loop haplotyping, Mangalicas were classified into two groups (Marincs et al. 2013). Animals in the first group carried major European haplotypes, while animals in the second group had Mangalica-specific haplotypes. In this respect, the Mangalicas imported to the USA and China, are the representatives of the first group, while Mangalicas 4S, 15F and 13V belong to the second group. We hypothesized that the two groups should have been evolved by different routes (Marincs et al. 2013).

It was previously shown (Marincs et al. 2013) that 37 of the examined 203 Mangalica and each of the 35 Turopolje individuals shared one major European D-loop haplotype (HAP08). In this respect, it is interesting that the two Turopolje pigs, included in this study, have HAP13. It is also important to note that, although HAP08 and HAP13 differ in only one SNP and are very closely related in a median-joint network of haplotypes (Marincs et al. 2013), the two HAP08 Blonde Mangalicas from the USA (Cannon et al. 2015) are in one clade with other HAP08 breeds and not with HAP13 Mangalicas (Figure 1). This is very likely due to the seven SNP differences between the USA Blonde and Swallow-belly Mangalicas (Supplementary Fig. S3), which are outside of the previously examined D-loop sequence used for haplotyping (Marincs et al. 2013). The phylogenetic position and the sequence differences between the USA Blonde and other Mangalicas indicate the possibility of introgression of other breeds into the progenitors of the USA Blonde Mangalicas.

Nevertheless, our study indicates that Turopolje pigs might be the closest relatives of a certain type of Mangalicas at the mitogenome level (Figure 1), which is interesting because the Turopolje breed was not named as a possible contributor to Mangalicas (Bökönyi 1974; Egerszegi et al. 2003). In the case of Mangalica, it was hypothesized that their ancestors, the Alföldi, Bakonyi and Szalontai pigs, were evolved from a much older pig race with the presumed origin from domesticated Slovenian pigs and wild boars and possibly from pigs brought to and kept in Pannonia province by the Romans (Egerszegi et al. 2003). The already extinct Serbian Sumadia (Sumadinka) and Syrmian breeds were also involved in the development of Mangalica (Bökönyi 1974; Egerszegi et al. 2003). Turopolje, named after the region where it emerged, is thought to be developed from some old races, such as Siska, Krskopoljski, black Slovenian and white South Austrian/North Croatian pigs (GENETICRESOURCES).

There are different possibilities to explain the relationship between the maternal lineages of Mangalica and Turopolje pigs. The first is a common wild boar ancestor since wild boars were domesticated in several centres around the World (Ramos-Onsins et al. 2014). The Siska pig, which is thought to be one of the direct descendants of wild boar and wild boar itself, was assumed to be amongst the ancestor of Turopolje and Mangalica, respectively (CEPIB; Egerszegi et al. 2003). Controversially, in ten present-day Hungarian wild boar samples the ANC-Cside ancient signature was found exclusively, while Mangalica and Turopolje haplotypes are associated with the ANC-Aside signature (Marincs et al. 2013).

The second putative common ancestors for Mangalica and Turopolje are the pigs kept by Romans in or brought to the provinces Pannonia (current Transdanubia and the Western Carpathian Basin) and Dalmatia (covering areas of present-day Slovenia, Croatia and Serbia). Regarding the possible Roman origin of the two breeds, the ANC-Aside signature was found in each examined Hungarian archaeological pig samples, which dated back to either Roman times or A.D., but in none of the Croatian archaeological pig samples, which dated back to B.C. (Larson et al. 2007). Though only a few samples were available in both cases and the Croatian samples were found in such geographic regions (Larson et al. 2007), which are not associated with the known geographic origin of the predecessors of the Turopolje and Mangalica breeds. Regarding the possibility of bringing the ancestors of Mangalica and Turopolje into the Carpathian Basin by Romans, it is interesting to note that the ANC-Aside ancient signature, being exclusively present in both Mangalicas and Turopolje, was more frequently found in Italian domesticated pigs, both modern and archaeological samples, than any other ancient D-loop signatures (Larson et al. 2007). The ANC-Aside signature was also more frequent in modern Italian wild boars but was not found amongst the eight archaeological Italian wild boar samples (Larson et al. 2007). This suggests that, if Romans had brought pigs to the Carpathian Basin, those might have been pigs domesticated outside Italy.

The third possible explanation of the relationship between the Mangalica and Turopolje breeds is a later-day admixture during the development of the breeds. Turopolje was spread along the Sava River as far as the Posavina region (the last refuge of the breed) of Slavonia (GENETICRESOURCES), where Turopolje could come into geographical vicinity and mate with the Syrmian and Sumadia pigs, which were described to be involved in the development of Mangalica (Molnár et al. 2013). Furthermore, the Turopolje breed also spread to the northern Podravina province of Slavonia and then further into south-west Hungary (ANSI), and thus the geographical distribution of Turopolje and Mangalica became overlapped to such a degree that they have had the opportunity to interbreed. Another indication for the admixture is that in a study involved only three breeds, Mangalica (from Austria and Serbia), Turopolje (from Austria and Croatia) and Black Slavonian (from Croatia), Black Slavonian populations were found to be more closely related either to Mangalica or to Turopolje (Druml et al. 2012). Black Slavonian might have been contributed to the development of the ancestors of Mangalica (Egerszegi et al. 2003) and Turopolje (GENETICRESOURCES), which can explain that observation.

The off-white curly hair of Turopolje (ANSI) resembles the curly coat of Mangalica and Sumadia, a contributor of Mangalica, and might be a sign of the relationship between these breeds. Apart from these three breeds, this very rare phenotype was only described in the Brazilian Canastrao breed, which might have common Mediterranean ancestors with Turopolje (Rhoad 1934), and the already extinct Lincolnshire Curly Coat breed (BPA).

## Conclusions

Here we presented the mitogenome of one individual of each of the three Hungarian Mangalica breeds from the Hungarian Mangalica Gene Reserve and compared them with that of other Mangalicas, of which mitogenome was sequenced in other countries. It was found that the mitogenomes of the examined Mangalicas, with the exception of two USA Blonde animals, are highly related to that of the Croatian Turopolje breed. In the case of animals with major European D-loop haplotypes, Roman origin, admixture, and common wild boar ancestors are all possible explanations for the relationship. However, it still remains mysterious that what might be the origin of the breed-specific D-loop haplotypes in very large number of Mangalicas.

## Supplementary Material

TMDN_A_1390415_Supplementary_Information.pdf
