# Supplementary material for: The full mitochondrial genomes of Mangalica pig breeds and their possible origin
Source: Mitochondrial DNA B Resour. 2017 Oct 17;2(2):730–4. doi: 10.1080/23802359.2017.1390415 (PMC7800509; doi:10.1080/23802359.2017.1390415)
Supplement: TMDN_A_1390415_Supplementary_Information.pdf [file TMDN_A_1390415_SM7999.pdf]

Supplementary material

The full mitochondrial genomes of Mangalica pig breeds and their possible origin

Krisztián Frank[1], János Molnár[1,2], Endre Barta, and Ferenc Marincs

Agricultural Biotechnology Center, National Agricultural Research and Innovation Centre, Szent-Györgyi Albert u. 4., Gödöllő, Hungary

Correspondence: Ferenc Marincs, PhD, Agricultural Biotechnology Center, National Agricultural Research and Innovation Centre, Szent-Györgyi Albert u. 4., H-2100 Gödöllő, Hungary. Tel: +36 28 526 225. E-mail: marincs.ferenc@abc.naik.hu

[1] These authors contributed equally
[2] Present address: Enviroinvest Ltd., Katona József u. 39., H-1137 Budapest, Hungary

E-mail addresses
KF: frank.krisztian@abc.naik.hu
JM: molnar.janos@envinvest.hu
EB: barta.endre@abc.naik.hu
FM: marincs.ferenc@abc.naik.hu

Supplementary Table S1. Mitogenomes used in this study.
References for Table S1.
Supplementary Figure S1. Phylogenetic tree of the mitogenomes in Table S1.
Supplementary Figure S2. Alignment of Mangalica mitogenomes.
Supplementary Figure S3. The number of SNP differences between Mangalica mitogenomes.
Supplementary Figure S4. D-loop haplotypes of the individual pigs in the European clade of the phylogenetic tree (Fig. 1), according to Marincs et al. (2013).

Supplementary Table S1. Mitogenomes used in this study.

Accession number	Country of sequence deposition	Origin	Breed	Reference
KF888634	China	Asian	Bama	Yang et al. 2016
KP126939	China	Asian	Bama xiang	Unpublished
EF545583	China	Asian	Bamei	Wu et al. 2007b
GQ220328	China	Asian	Banna	Unpublished
KP789021	China	Asian	Bearded pig	Unpublished
AY574045[1]	South Korea	European	Berkshire	Unpublished
KC505410[1]	China	European	Berkshire	Unpublished
KP765602[1]	China	European	Berkshire	Unpublished
EF545590	China	Asian	Bihu	Wu et al. 2007b
MF183222	Hungary	European	Blond Mangalica	This study
JN601067	USA	European	Blond Mangalica	Cannon et a. 2015
JN601066	USA	European	Blond Mangalica	Cannon et a. 2015
KP765603	China	Asian	Chenghua	Unpublished
KM250424	China	Asian	Congjiang	Ran et al. 2016
KP294522	China	Asian	Dabieshan	Unpublished
GQ220329	China	Asian	Dahe	Unpublished
KM200762	China	Asian	Dahuabai	Unpublished
KF472179	China	Asian	Daweizi	Xu et al. 2015a
KM044240	China	Asian	Diannan	Wang et al. 2016
KJ737417	China	Asian	Ding Yuan	Unpublished
MF183225	Hungary	European	Duroc	This study
AY337045	South Korea	European	Duroc	Unpublished
KF767444	China	European	Duroc	Unpublished
FJ236997	Spain	European	Duroc	Unpublished
FJ236996	Spain	European	Duroc	Unpublished
AJ002189	Sweden	European	Duroc	Ursing and Arnason 1998
KM101042	China	Asian	Guanling	Unpublished
AY574046	South Korea	European	Hampshire	Unpublished
KJ737418	China	Asian	Hang	Unpublished
KJ737419	China	Asian	Huai	Unpublished
KJ737420	China	Asian	Huo shou	Unpublished
EF545587	China	Asian	Huzu	Wu et al. 2007b
EF545588	China	Asian	Huzu	Wu et al. 2007b
FJ236991	Spain	European	Iberian	Unpublished
FJ236992	Spain	European	Iberian	Unpublished
FJ236993	Spain	European	Iberian	Unpublished
FJ236994	Spain	European	Iberian	Unpublished
FJ236995	Spain	European	Iberian	Unpublished
EU117375	Spain	European	Iberian	Unpublished
AY334492	South Korea	Asian	Jeju	Unpublished
DQ334861	South Korea	Asian	Jeju	Unpublished

DQ334860	South Korea	Asian	Jeju	Unpublished
DQ274110	South Korea	Asian	Jeju	Unpublished
KC469586	China	Asian	Jinhua	Yu et al. 2013
KJ737421	China	Asian	Laiwu	Unpublished
KJ720205	China	Asian	Laiwu	Unpublished
AF034253	Taiwan	European	Landrace	Lin et al. 1999
NC000845	Taiwan	European	Landrace	Lin et al. 1999
KM101043	China	Asian	Lantang	Ran et al. 2016
KC250274	China	Asian	Lantang	Yu et al. 2013
EF375877	Taiwan	Asian	Lanyu Type I	Wu et al. 2007a
DQ972936	Taiwan	Asian	Lanyu Type II	Wu et al. 2007a
AY574048[1]	South Korea	European	Large White	Unpublished
KC250275	China	European	Large White	Yu et al. 2013
KP257598	China	Asian	Liangshan	Unpublished
KP681242	China	Asian	Lingao	Unpublished
KJ737422	China	Asian	Longlin	Unpublished
KM433673	China	Asian	Longlin	Hu et al. 2016
KJ737423	China	Asian	Luchuan	Unpublished
KP126954	China	Asian	Luchuan	Zhang et al. 2016
KM275217	China	Asian	Luchuan	Unpublished
KJ746662	China	Asian	Ma shen	Unpublished
KJ746666	China	European	Mangalica	Unpublished
KM998967	China	Asian	Meishan	Unpublished
JN601071	USA	Asian	Meishan	Unpublished
JN601070	USA	Asian	Meishan	Unpublished
KF971862	China	Asian	Min	Unpublished
KJ746663	China	Asian	Neijiang	Unpublished
KC505406	China	Asian	Neijiang	Unpublished
KF472178	China	Asian	Ningxiang	Xu et al. 2015c
DQ466081	China	Asian	Nuogu	Unpublished
KJ746664	China	Asian	Penzhou	Unpublished
KC505407	China	Asian	Penzhou	Unpublished
KC469587	China	European	Pietrain	Yu et al. 2013
KF660222	China	Asian	Qianshao	Unpublished
EF545582	China	Asian	Qingping	Wu et al. 2007b
EF545581	China	Asian	Qingping	Wu et al. 2007b
KP257599	China	Asian	Qingyu	Unpublished
MF183223	Hungary	European	Red Mangalica	This study
KM044239	China	Asian	Rongchang	Wang et al. 2016
EF545567	China	Asian	Saba	Wu et al. 2007b
KM094194	China	Asian	Sandu	Wang et al. 2016
KF472177	China	Asian	Shaziling	Xu et al. 2015d
MF183224	Hungary	European	Swallow-belly Mangalica	This study
JN601069	USA	European	Swallow-belly Mangalica	Cannon et a. 2015
JN601068	USA	European	Swallow-belly Mangalica	Cannon et a. 2015

KF601700	China	Asian	Taoyuan	Unpublished
DQ534707	Taiwan	Asian	Taoyuan	Unpublished
KM073256	China	Asian	Tibetan	Unpublished
KC493612	China	Asian	Tibetan	Unpublished
KC493611	China	Asian	Tibetan	Unpublished
KC493610	China	Asian	Tibetan	Unpublished
KC493609	China	Asian	Tibetan	Unpublished
KC493608	China	Asian	Tibetan	Unpublished
KC493607	China	Asian	Tibetan	Unpublished
JN601073	USA	European	Turopolje	Cannon et a. 2015
JN601072	USA	European	Turopolje	Cannon et a. 2015
EF545577	China	Asian	Wei	Wu et al. 2007b
KP681244	China	Asian	Wenchang	Unpublished
KP765604	China	Asian	Wuhuang	Unpublished
KC505408	China	Asian	Wujin	Unpublished
KM259826	China	Asian	Wuyi	Xiao et al. 2016
KF767443	China	Asian	Wuzhishan	Unpublished
KP681243	China	Asian	Wuzhishan	Unpublished
KJ909516	China	Asian	Wuzhishan	Unpublished
KC250273	China	Asian	Xiang	Yu et al. 2013
EF545593	China	Asian	Xiang	Wu et al. 2007b
KJ746665	China	Asian	Ya cha	Unpublished
KC505409	China	Asian	Yanan	Unpublished
EF545589	China	Asian	Yimenghei	Wu et al. 2007b
KF752550[1]	China	European	Yorkshire	Xu et al. 2016
KF569218	China	European	Yorkshire	Xu et al. 2015b
JN601075[1]	USA	European	Yorkshire	Cannon et a. 2015
JN601074[1]	USA	European	Yorkshire	Cannon et a. 2015
EF545576	China	Asian	Zang	Wu et al. 2007b

[1] Phylogenetic analysis placed these individuals into the Asian clade, indicating introgression of Asian mtDNA into these animals.

References for Table S1.

Cannon MV, Brandebourg TD, Kohn MC, Đikić D, Irwin MH, Pinkert CA. (2015). Mitochondrial DNA sequence and phylogenetic evaluation of geographically disparate *Sus scrofa* breeds, Anim Biotechnol 26:17-28.

Hu X, Xiao D, Li W. (2016). The complete sequence of the mitochondrial genome of Longlin pig (*Sus scrofa*). Mitochondrial DNA Part A 27:1736-1737.

Lin CS, Sun YL, Liu CY, Yang PC, Chang LC, Cheng IC, Mao SJ, Huang MC. (1999). Complete nucleotide sequence of pig (*Sus scrofa*) mitochondrial genome and dating evolutionary divergence within Artiodactyla. Gene 236:107-114.

Ran ML, Wang MF, Yang AQ, Li Z, Chen B. (2016). The complete mitochondrial genome of Congjiang miniature pig (*Sus scrofa*). Mitochondrial DNA Part A 27:1787-1788.

Ursing BM, Arnason U. (1998). The complete mitochondrial DNA sequence of the pig (*Sus scrofa*). J Mol Evol 47:302 306.

Wang LY, Xu D, Xiao DF, Ma HM. (2016). The complete mitochondrial genome sequence of Diannan small-ear pig (*Sus scrofa*). Mitochondrial DNA Part A 27:1309-1310.

Wu CY, Jiang YN, Chu HP, Li SH, Wang Y, Li YH, Chang Y, Ju YT. (2007a). The type I Lanyu pig has a maternal genetic lineage distinct from Asian and European pigs. Anim Genet 38:499-505.

Wu GS, Yao YG, Qu KX, Ding ZL, Li H, Palanichamy MG, Duan ZY, Li N, Chen YS, Zhang YP. (2007b). Population phylogenomic analysis of mitochondrial DNA in wild boars and domestic pigs revealed multiple domestication events in East Asia. Genome Biol 8:R245.

Xiao D, Hu X, Chen Y, Gong Z, Chen L. (2016). The complete sequence of mitochondrial genome of Wuyi Black pig (Sus Scrofa). Mitochondrial DNA Part A 27:1585-1586.

Xu D, He CQ, Li QH, He J, Ma HM. (2015a). The complete mitochondrial genome of the Daweizi pig. Mitochondrial DNA 26:640-641.

Xu D, He CQ, He J, Yang H, Ma HM. (2015b). Mitochondrial DNA sequence of the hybrid of Duroc (♂) × [Landrace (♂) × Yorshire (♀)] pig. Mitochondrial DNA 26:682-683.

Xu D, Li QH, He CQ, Chai YL, Ma HM. (2015c). The complete mitochondrial genome of the Ningxiang pig. Mitochondrial DNA 26:623-624.

Xu D, Li QH, He CQ, Wang LY, Ma HM. (2015d). The complete mitochondrial genome of the Shaziling pig. Mitochondrial DNA 26:619-620.

Xu D, Yang H, Ma H. (2016). The complete mitochondrial genome of the Yorkshire pig (*Sus scrofa*). Mitochondrial DNA Part A 27:641-642.

Yang H, Xu XL, Xu D, Ma HM, Li LL. (2016). The complete sequence of mitochondrial genome of Bama miniature pig (*Sus scrofa*). Mitochondrial DNA Part A 27:238-239.

Yu G, Xiang H, Wang J, Zhao X. (2013). The phylogenetic status of typical Chinese native pigs: analyzed by Asian and European pig mitochondrial genome sequences. J Anim Sci Biotechnol 8:9.

Zhang Y, Xie Z, Deng X, Xie Z, Liu J, Xie L, Luo S, Huang L, Huang J, Zeng T, Wang S. Mitochondrial genome of the Luchuan pig (*Sus scrofa*). (2016). Mitochondrial DNA Part A 27:4139-4141.

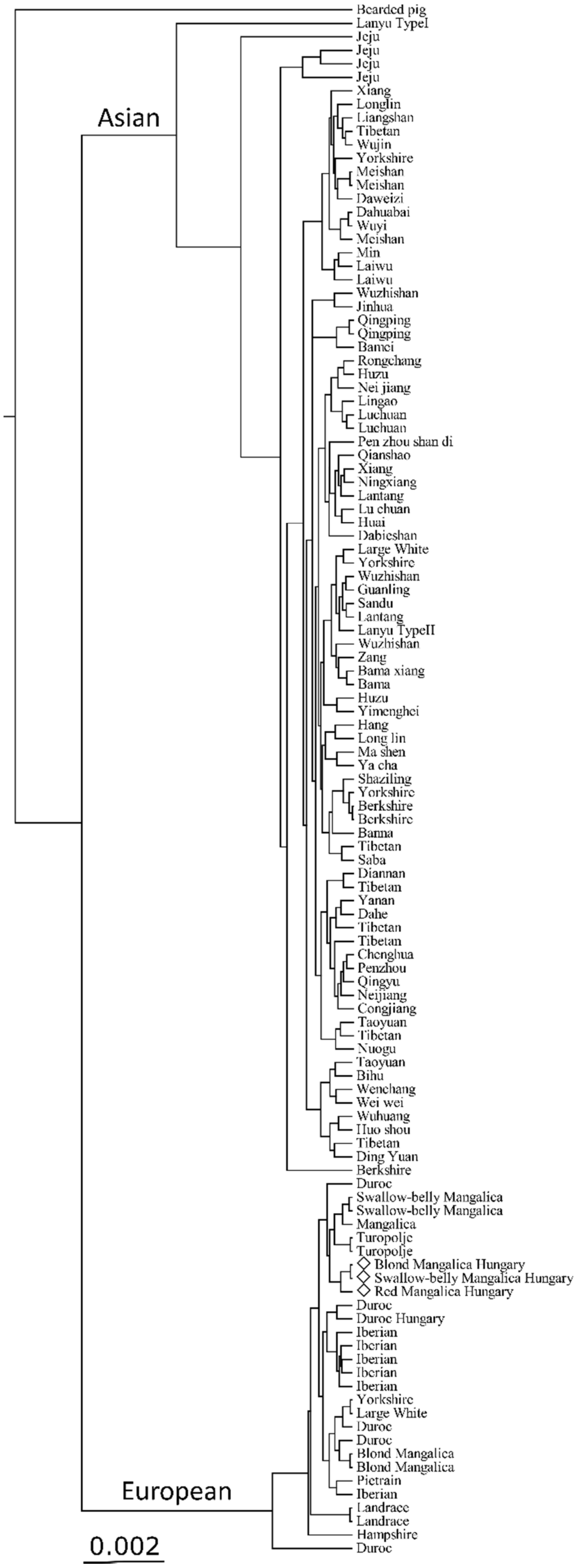

Supplementary Figure S1. Phylogenetic tree of the mitogenomes in Table S1.

The genome sequenced Mangalica individuals (Molnár et al. 2014) are labelled by a diamond. Scale bar represents branch length (substitution per site)

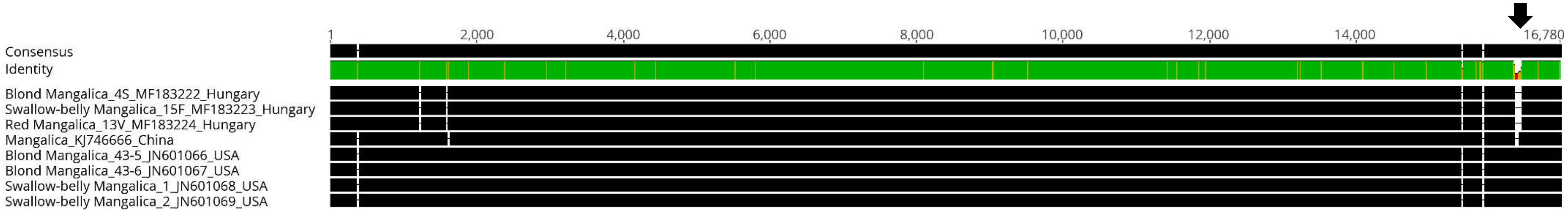

Supplementary Figure S2. Alignment of Mangalica mitogenomes.

Indels are represented by vertical white lines in the aligned sequences. Black arrow indicates the larger 90/50 bp indel.

	Blond Mangalica_4S_MF183222_Hungary	Swallow-belly Mangalica_15F_MF183223_Hungary	Red Mangalica_13V_MF183224_Hungary	Mangalica_KJ746666_China	Blond Mangalica_43-5_JN601066_USA	Blond Mangalica_43-6_JN601067_USA	Swallow-belly Mangalica_1_JN601068_USA	Swallow-belly Mangalica_2_JN601069_USA
**Blond Mangalica_4S_MF183222_Hungary**		0	7	33	19	19	16	16
**Swallow-belly Mangalica_15F_MF183223_Hungary**			7	33	19	19	16	16
**Red Mangalica_13V_MF183224_Hungary**				32	18	18	15	15
**Mangalica_KJ746666_China**					24	24	17	17
**Blond Mangalica_43-5_JN601066_USA**						0	7	7
**Blond Mangalica_43-6_JN601067_USA**							7	7
**Swallow-belly Mangalica_1_JN601068_USA**								0
**Swallow-belly Mangalica_2_JN601069_USA**								

Supplementary Figure S3. The number of SNP differences between Mangalica and Turopolje mitogenomes.

In the matrix, numbers and colored scales indicate the number of SNP differences between the individuals.

				Reference sequence position									
**Breed**	**ID**	**Country**	**NCBI No.**	**15558**	**15615**	**15674**	**16682**	**15706**	**15714**	**15758**	**Haplotype (Marincs et al. 2013)**	**Ancient signature (Larson et al. 2007)**	**Reference (Table S1)**
**Duroc**	D2	Spain	FJ236997	T	C	T	G	-	C	C	**HAP13**	ANC-Aside	Unpublished
**Swallow-belly Mangalica**	2	USA	JN601069	T	C	T	G	-	C	C	**HAP13**	ANC-Aside	Cannon et al. 2015
**Swallow-belly Mangalica**	1	USA	JN601068	T	C	T	G	-	C	C	**HAP13**	ANC-Aside	Cannon et al. 2015
**Mangalica**		China	KJ746666	T	C	T	G	-	C	C	**HAP13**	ANC-Aside	Unpublished
**Turopolje**		USA	JN601072	T	C	T	G	-	C	C	**HAP13**	ANC-Aside	Cannon et al. 2015
**Turopolje**		USA	JN601073	T	C	T	G	-	C	C	**HAP13**	ANC-Aside	Cannon et al. 2015
**Blonde Mangalica**	4S	Hungary	MF183222	T	C	C	A	-	C	C	**HAP16**	ANC-Aside	This study
**Swallow-belly Mangalica**	15F	Hungary	MF183224	T	C	C	A	-	C	C	**HAP16**	ANC-Aside	This study
**Red Mangalica**	13V	Hungary	MF183223	T	C	C	G	-	C	C	**HAP45**	ANC-Aside	This study
**Reference seq (Duroc)**		Sweden	AJ002189	A	T	T	G	C	T	T	**HAP149**	ANC-Cside	Ursing and Arnason 1998
**Duroc**	1172D	Hungary	MF183225	A	T	T	G	C	C	T	**NEW**	ANC-Cside	This study
**Iberian**		Spain	EU117375	A	T	T	G	-	T	C	**NEW**	ANC-Finnish41	Unpublished
**Iberian**	H2	Spain	FJ236991	A	T	T	G	-	C	C	**HAP07**	ANC-Cside	Unpublished
**Iberian**	H4	Spain	FJ236993	A	T	T	G	-	C	C	**HAP07**	ANC-Cside	Unpublished
**Iberian**	H3	Spain	FJ236992	A	T	T	G	-	C	C	**HAP07**	ANC-Cside	Unpublished
**Iberian**	H6	Spain	FJ236995	A	T	T	G	-	C	C	**HAP07**	ANC-Cside	Unpublished
**Yorkshire**		China	KF569218	T	T	T	G	-	T	C	**HAP44**	ANC-Aside	Xu et al. 2015b
**Large White**		China	KC250275	T	T	T	G	-	T	C	**HAP44**	ANC-Aside	Yu et al. 2013
**Duroc**	D1	Spain	FJ236996	T	T	T	G	-	T	C	**HAP44**	ANC-Aside	Unpublished
**Duroc**		China	KF767444	T	T	T	G	-	C	C	**HAP08**	ANC-Aside	Unpublished
**Blonde Mangalica**	43-5	USA	JN601066	T	T	T	G	-	C	C	**HAP08**	ANC-Aside	Cannon et al. 2015
**Blonde Mangalica**	43-6	USA	JN601067	T	T	T	G	-	C	C	**HAP08**	ANC-Aside	Cannon et al. 2015
**Pietrain**		China	KC469587	T	T	T	G	-	C	C	**HAP08**	ANC-Aside	Yu et al. 2013
**Iberian**	H5	Spain	FJ236994	T	T	T	G	-	C	C	**HAP08**	ANC-Aside	Unpublished
**Landrace**		Taiwan	AF034253	T	T	T	G	-	C	C	**HAP08**	ANC-Aside	Lin et al. 1999
**Landrace**		Taiwan	NC000845	T	T	T	G	-	C	C	**HAP08**	ANC-Aside	Lin et al. 1999
**Hampshire**		South Korea	AY574046	T	T	T	G	-	C	C	**HAP08**	ANC-Aside	Unpublished
**Duroc**		South Korea	AY337045	T	T	T	G	-	C	C	**HAP08**	ANC-Aside	Unpublished

Supplementary Figure S4. D-loop haplotypes of the individual pigs in the European clade of the phylogenetic tree (Fig. 1), according to Marincs et al. (2013).

The order of the individuals from the top to the bottom is the same as in Fig. 1 in the main text. The same haplotypes are highlighted by the same color (also see Fig. 1 in the main text).
